# 

**Published:** 1868-12-01

**Authors:** 


### *Books Received.*

Transactions of the American Medical Association, Instituted 1847., Vol. XIX, Philadelphia; printed for the Association. Collins, printer, 705 Jayne Street, 1868 Pp. Notice next month.

THE TWELFTH EDITION OF ELLIS'S MEDICAL FORMULARY, heretofore frequently mentioned in THE JOURNAL, has come to hand. Pp. 374. W. B. Keen & Co., Chicago.

This is really a *new* edition of a well-known work. Many formulæ of little value have been omitted, the table of doses has been carefully corrected, and new formulæ of value have been introduced. The new classes of anti-emetics and disinfectants, and also the use of atomized fluids receive careful attention. A new feature is introduced in a full index of diseases, with references to the members of principal ingredients of the formulæ applicable to each.

A HAND BOOK OF VACCINATION, by Edward C. Seaton, M.D., Medical Instructor to the Privy Council. Philadelphia: J. B. Lippincott & Co., 1868. Pp. 383.

A complete *resume* of what is known on the subject. We shall refer to it hereafter.

A TREATISE ON PHYSIOLOGY AND HYGIENE, for Schools, Families and Colleges. By J. C. Dalton, M.D., Prof. of Physiology in the College of Physicians and Surgeons, New York. With Illustrations. New York: Harper & Brothers, Publishers. London: Sampson Low, Son & Marston. 1868. Pp. 399.

The professional reputation of the author of this book is fully sustained in this most dangerous of all books to write. It is every way reliable, compact, clear, fully adapted to the purpose set forth in its title.

THE OPIUM HABIT. With Suggestions as to the Remedy. New York: Harper & Brothers, Publishers, Franklin Square. 1868. Pp. 235. Notice next No.

In the next number also we shall mention our exchanges, particularly those non-professional. We regret the paucity of our pages, and only regret that we had not double the room to contain the *crowd* of good things that are now pressing upon our pages. An immensity has to go over to subsequent issues.

Constipated Bowels: The Various Causes, and the Different Means of Cure. By S. B. Birch, M.D., Member of the Royal College of Physicians of London, etc., etc., etc. From the third London edition. Pp. 181. Philadelphia: Lindsay & Blakiston. 1868. Price, $1.25, at Keen & Co.'s

A very creditable monograph, and well worth perusal. The author is evidently a man of practical common sense. The name of the publishers is sufficient guaranty for the style of the publication.

